# The MAP3K7-mTOR Axis Promotes the Proliferation and Malignancy of Hepatocellular Carcinoma Cells

**DOI:** 10.3389/fonc.2019.00474

**Published:** 2019-06-04

**Authors:** Jin-Shiung Cheng, Wei-Lun Tsai, Pei-Feng Liu, Yih-Gang Goan, Chih-Wen Lin, Ho-Hsing Tseng, Cheng-Hsin Lee, Chih-Wen Shu

**Affiliations:** ^1^Division of Gastroenterology and Hepatology, Department of Internal Medicine, Kaohsiung Veterans General Hospital, Kaohsiung, Taiwan; ^2^School of Medicine, National Yang-Ming University, Taipei, Taiwan; ^3^Department of Medical Education and Research, Kaohsiung Veterans General Hospital, Kaohsiung, Taiwan; ^4^Division of Thoracic Surgery, Department of Surgery, Kaohsiung Veterans General Hospital, Kaohsiung, Taiwan; ^5^Division of Gastroenterology and Hepatology, E-Da Dachang Hospital, I-Shou University, Kaohsiung, Taiwan; ^6^School of Medicine for International Students, I-Shou University, Kaohsiung, Taiwan

**Keywords:** kinome siRNA library screening, MAP3K7, proliferation, migration, invasion, mTOR, prognosis

## Abstract

Targeted therapy is currently limited for patients with hepatocellular carcinoma (HCC) due to the lack of suitable targets. Kinases play pivotal roles in many cellular biological processes, whereas dysregulation of kinases may lead to various diseases, particularly cancer. However, the role of kinases in HCC malignancy remains unclear. In this study, we employed a kinome small interfering RNA (siRNA) library, comprising 710 kinase-related genes, to screen whether any kinases were essential for cell proliferation in various HCC cell lines. Through a kinome siRNA library screening, we found that MAP3K7 was a crucial gene for HCC cell proliferation. Pharmacological or genetic ablation of MAP3K7 diminished the growth, migration, and invasion of HCC cells, including primary HCC cells. Stable knockdown of MAP3K7 attenuated tumor formation in a spheroid cell culture model and tumor xenograft mouse model. In addition, silencing MAP3K7 reduced the phosphorylation and expression of mammalian target of rapamycin (mTOR) in HCC cells. MAP3K7 expression was positively correlated with mTOR expression in tumors of patients with HCC. Higher co-expression of MAP3K7 and mTOR was significantly associated with poor prognosis of HCC. Taken together, our results revealed that the MAP3K7-mTOR axis might promote tumorigenesis and malignancy, which provides a potential marker or therapeutic target for HCC patients.

## Introduction

Hepatocellular carcinoma (HCC) is the one of the most prevalent malignant tumors and major causes of cancer-related death worldwide. Curative treatments for patients with HCC include surgical resection, liver transplantation, and radiofrequency ablation-percutaneous ethanol injection (1, 2); however, only patients with early-stage HCC are eligible for curative treatments. Systemic chemotherapy is challenging for patients with compromised liver function, which is present in most HCC patients (3). Sorafenib was the first targeted drug to be clinically approved for use in patients with advanced HCC (4–6). The major target of sorafenib is the serine-threonine kinase Raf-1, which is involved in the Ras/Raf/MEK/mitogen-activated protein kinase signaling (MAPK) cascade (7). Although sorafenib efficiently inhibits the activity of Raf-1 at a very low dose (half maximal inhibitory concentration value of 6 nM) (8, 9), an increasing number of reports have shown that sorafenib also inhibits several receptor tyrosine kinases, such as vascular endothelial growth factor receptor (VEGFR) 1, 2, and 3, platelet-derived growth factor receptor (PDGFR), and fibroblast growth factor receptor (9–11). Moreover, sorafenib showed limited survival benefits in large, randomized phase III studies, with a very low response rate (2–3%) (12, 13), likely due to molecules involved in epithelial–mesenchymal transition, cancer stemness, and the tumor microenvironment for drug resistance (14). Effective therapeutic targets for HCC are currently unavailable, demonstrating an urgent need to identify suitable molecules to overcome this pressing issue.

Kinases are crucial upstream regulators of the signaling pathways required for homeostasis in normal cells. Dysregulation of oncogenic kinases by either overexpression or overactivation is highly associated with tumorigenesis, migration, invasion, and drug resistance (15–17). In addition to the involvement of the aforementioned kinases in HCC, many kinases are reportedly associated with tumor progression in HCC. Aurora kinase A (AURKA) is associated with cancer metastasis and stemness in HCC cells, particularly in TP53-mutated HCC cells (18, 19). Mammalian target of rapamycin (mTOR) is overexpressed in 50% of HCCs and its expression is correlated with poor differentiation and prognosis (20). Focal adhesion kinase upregulates the proto-oncogenes EZH2 and H3K27me3 for HCC cell growth (21). These genes are concomitantly expressed at a higher level in HCC than in non-tumor liver. *Dnajb1–Prkaca* gene fusion activates PRKACA kinase to promote the tumorigenesis of fibrolamellar HCC in mice (22). Moreover, annexin A3 activates JNK for the growth of HCC cells, particularly of CD133^+^ liver cancer stem cells (23). Although many kinases play pivotal roles in signaling pathways that are associated with HCC tumorigenesis, no inhibitor targeting these kinases is largely beneficial for patients with HCC, indicating that little is known about kinases in HCC therapy.

In this study, we employed a kinome siRNA library to identify potential kinases required for the survival of HCC cells. We found that mitogen-activated protein kinase kinase kinase 7 (MAP3K7) appears to be essential for the growth and metastatic characteristics of HCC cells. Genetic and pharmacological targeting of MAP3K7 attenuated tumor cell growth in spheroid cell culture and a xenograft mouse model. Additionally, MAP3K7 expression was positively correlated with mTOR expression, and high co-expression of MAP3K7 and mTOR was associated with poor survival in patients with HCC. Taken together, our results suggest that MAP3K7 might be a potential target for the future development of targeted therapy for HCC.

## Materials and Methods

### Cell Culture, Transfection, and Stable Selection

SK-HEP-1, Huh7 (Huh7.5.1), Hep3B, and HA22T HCC cancer cell lines (American Type Culture Collection, Manassas, VA) were cultured in Dulbecco's modified Eagle's medium (DMEM) with 10% fetal bovine serum (FBS), 100 μg/mL streptomycin, 100 IU penicillin, and 1% L-glutamine at 37°C in 5% CO_2_ and 95% air. For primary cell culture, HCC 71T and 89T cells were isolated by Dr. Hung-Wei Pan from surgically resected HCC patients, which was approved by Kaohsiung Veterans General Hospital (IRB protocol: VGHKS13-CT3-009). The primary HCC cells were cultured in DMEM/F12 (1:1) supplemented with basic fibroblast growth factor (15 ng/mL), epidermal growth factor (20 ng/mL), L-glutamine (2 mM/L), insulin growth factor (4 U/L), and B27 supplement (1:50). For spheroid cell culture, HCC cells were seeded at a density of 2.0 × 10^4^ cells/well in 24-well NanoCulture plates (1.9 cm^2^, SCIVAX Corporation, Kanagawa, Japan). The cells were cultured for 7 days until spheroid formation (diameter > 0.1 mm). For gene knockdown with siRNA, the cells were transfected with 5 nM scramble siRNA or siRNA against Src kinase (6714, Dhamacon, Lafayette, CO) for 72 h using Lipofectamine RNAiMAX (13778-150, Invitrogen, Carlsbad, CA). For lentivirus infection, HEK293T cells were seeded into 6-well plates and transfected with 2 μg scramble short hairpin RNA (shRNA) or shRNA against MAP3K7 (TRCN0000195383), AURKA (TRCN0000010533), polo-like kinase 1 (PLK1) (TRCN0000121325), or phosphoinositide-3-kinase-catalytic-alpha (PIK3CA) (TRCN0000196795) using 1 μL Lipofectamine 2000 (11668027, Invitrogen) for 16 h. The transfected cells were washed with medium and incubated for 48 h. The cell debris was removed with 0.45 μm filter and the supernatant was used to infect HCC cells with 10 μg/mL polybrene (TR-1003-G, Sigma-Aldrich, USA) for 24 h. The cells were then maintained in culture medium with 1 μg puromycin and the medium was refreshed every 3 days to obtain stable cell lines. The knockdown efficiency was confirmed by real-time PCR.

### Real-Time PCR

The mRNA level of each gene in the cells was amplified SYBR Green Master Mix (4385612, Applied Biosystems) and analyzed by a StepPnePlus system (Applied Biosystems, USA) as described previously (24). The primer sequences used for gene expression were shown as followings: MAP3K7 forward 5′- CCGGTGAGATGATCGAAGCC-3′ and reverse 5′- GCCGAAGCTCTACAATA AACGC-3′. GAPDH forward 5′-TGCACCACCAACTGCTTAGC-3′ and reverse 5′-GGCATGGACTGTGGTCAT−3′ (as a normalized control). The primer sequences for the other genes will be provided upon request.

### Cell Proliferation Assay

For cell proliferation assays with siRNA screening, SK-HEP-1 cells harboring a luciferase plasmid (2.0 × 10^3^ cells/40 μL) were seeded into each well of a 384-well white plate containing 10 nM scramble siRNA or kinome siRNA library (2127 siRNA for 709 genes, A30079, Thermo Fisher Scientific, Waltham, MA) and RNAiMAX (13778-150, Invitrogen) for 72 h. The cells were mixed with Cell-Titer Glo (G7572, Promega, Madison, WI), and the luminescent signal was monitored to reflect cell proliferation (as measured by the ATP level). In addition, cells treated with (5Z)-7-oxozeaenol (499610, Merck, Kenilworth, NJ) for 24 h were mixed with Cell-Titer Glo (G7572, Promega) and luminescent signals were read with a Fluoroskan Ascent FL Reader (Thermo Fisher Scientific). For clonogenic assays, HCC cells were seeded in 12-well plates at a density of 3.0 × 10^3^ cells/well, as described previously (25). The culture medium was replaced every 3 days for 14 days until colony formation. The cell colonies were fixed in 2% paraformaldehyde, stained with crystal violet (0.25% w/v), and counted to determine cytotoxicity.

### Cell Mobility Assay

For the wound healing assay, 1.5 × 10^5^ silenced cells in 140 μL DMEM were seeded in culture inserts (IBIDI, Inc., Planegg, Germany) for 8–16 h. Subsequently, the culture inserts were removed for 17–18 h and the cells were fixed to measure migration distance. Transwell invasion assays were performed using 8-μm pore inserts (Greiner Bio-One, Stroud, UK) as described previously (26). Briefly, the silenced cells were seeded into the top chamber of 0.5% Matrigel-coated Transwell plates in 300 μL DMEM containing 1% FBS. After invasion, the cells were fixed and stained with 0.1% crystal violet. The invaded cells were observed under a microscope at a magnification of ×200 and quantified with ImageJ software.

### Western Blot Analysis

The cells were lysed with RIPA buffer as described previously (27), and proteins were separated by sodium dodecyl sulfate-polyacrylamide gel electrophoresis and transferred onto nitrocellulose membranes. The membranes were blocked with bovine serum albumin and incubated with the following primary antibodies at 4°C overnight: anti-MAP3K7 (4505, Cell Signaling Technology, Danvers, MA), anti-phosphorylated (p)-mTOR (Ser2448) (5536, Cell Signaling Technology), anti-mTOR (2983, Cell Signaling Technology), anti-p-AMPK (5536, Cell Signaling Technology), anti-AMPK (2532, Cell Signaling Technology), anti-p-Ser792-raptor (2083, Cell Signaling Technology), anti-raptor (2280, Cell Signaling Technology), and anti-ACTB (A5441, Sigma-Aldrich, St. Louis, MO). The proteins were probed with IRDye-800 or−680 secondary antibodies (LI-COR, Lincoln, NE) at room temperature for 1–2 h and scanned to analyze protein expression with an Odyssey® Imaging System (LI-COR).

### Tumor Xenografts

Five-week-old immunodeficient mice (nu/nu female) were purchased and acclimated for 5 days prior to tumor implantation. SK-HEP-1 cells (2.0 × 10^6^) stably harboring scramble shRNA or shRNA against MAP3K7 were mixed with Matrigel for implantation into the mice. Tumor size was measured every 3–4 days and calculated by the formula 0.5 × (larger diameter) × (smaller diameter)^2^. All animal experiments were approved by the Institutional Animal Care and Use Committee at Kaohsiung Veterans General Hospital. Tumors were resected from sacrificed mice, weighed, and fixed in 10% paraformaldehyde for immunohistochemical analysis.

### Immunohistochemistry and Scoring

As the retrospective cohort for this study, tissue microarrays for HCC patients were purchased from US Biomax for use in determining the correlation between kinase hits and prognosis. Colorectal tissues were analyzed by immunohistochemical staining: the tissue sections were stained with an anti-MAP3K7 antibody (ab109526, Abcam, Cambridge, UK), followed by a horseradish peroxidase-conjugated secondary antibody to observe protein levels in both the tumor and adjacent normal cells. All tumor cells within each microscopic field were counted for the Allred scoring system, which is based on the sum of a proportion score and an intensity score. The estimated average staining intensity was scored as: 0 (all cells negative), 1+ (weak expression), 2+ (moderate expression), and 3+ (strong expression). The proportion was scored as: 0 (negative), 1 (1%), 2 (1–10%), 3 (10–33%), 4 (33–66%), and 5 (66–100%). In the subsequent data analysis, a kinase expression level score < 5 was considered “low,” whereas a score >5 was categorized as “high.”

### Statistical Analysis

The data are reported as the mean ± standard error of the mean (SEM) from three independent experiments. The results of colony formation, migration and invasion were performed by a non-parametric 2-tailed Student's *t*-test. The data were analyzed using analysis of variance (ANOVA) with Tukey's *post-hoc* test to analyze xenografted tumor size and gene expression in the HCC patient dataset obtained from The Cancer Genome Atlas (TCGA) database (https://cancergenome.nih.gov/). The Wilcoxon signed-rank test was used to evaluate the different levels of kinase between the tumor and corresponding tumor adjacent normal tissue. The cutoff for each gene or protein expression level was according to receiver operating characteristic (ROC) curve to divide it into high and low group. Cumulative survival curves were estimated using the Kaplan-Meier method. Univariate and multivariate Cox proportional hazards models were used for crude and adjusted hazard ratios, respectively. A *p* < 0.05 (2-sided) was considered significant.

## Results

### Kinome-Wide Screening for Kinases Required for HCC Cell Proliferation

**Figure 1:**
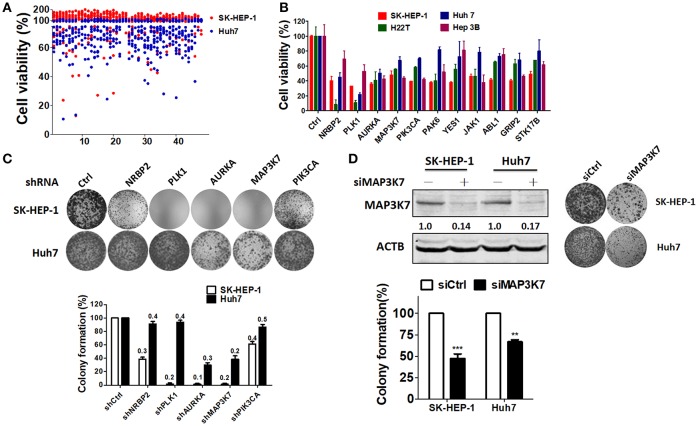
Kinome siRNA library screening for kinases required for HCC cell proliferation. **(A)** Hep3B and Huh7 cells were seeded in 384-well plates containing the kinome siRNA library (5 nM pooled siRNA for each gene) for 72 h. Cell proliferation was determined by a CellTiter Glo assay. **(B)** siRNAs against the top 11 ranked kinase hits were transfected into SK-HEP-1, HA22T, Huh7, and Hep3B cells for confirmation with a cell proliferation assay. **(C)** Stable knockdown with shRNA against NRBP2, PLK1, AURKA, MAP3K7, or PIK3CA in SK-HEP-1 and Huh7 cells was performed, and their effects were tested with a colony formation assay. Knockdown efficiency was examined by real-time PCR as indicated in each bar. **(D)** Knockdown efficiency in the MAP3K7 protein level of HCC cells was validated by immunoblotting. Inhibition of cell proliferation by siRNA against MAP3K7 in HCC cells was verified by a colony formation assay and quantified in the lower panel from three independent experiments. ^**^p < 0.01; ^***^p < 0.001.

Although hepatitis B virus (HBV) is one of the major risk factors for HCC (28), the aim of the present study was to identify a suitable kinase as a therapeutic target for most types of HCC, and not certain types of HCC. Thus, to evaluate if any kinases are required for HCC cell proliferation, two HBV-negative HCC cell lines (SK-HEP-1 and Huh7) (29) were transfected with a kinome siRNA library to determine the effect of each gene on HCC cell proliferation (Figure 1A). The top 11 ranked hits revealed that silencing these genes inhibited cell proliferation in SK-HEP-1 and Huh7 cells. Pooled siRNAs against these genes were transfected into HBV-negative (SK-HEP-1 and Huh7) and -positive (HA22T and Hep3B) HCC cell lines, to validate whether the kinases were essential for cell proliferation in different types of HCC cells (Figure 1B). The suppressive effects of siRNA against the top five genes on cell proliferation were consistent in the various HCC cell lines examined. Stable knockdown of these genes effectively diminished gene expression and colony formation in SK-HEP-1 cells, whereas only gene knockdown of AURKA and MAP3K7 inhibited colony formation in Huh7 cells (Figure 1C). The role of AURKA in HCC has been reported previously (18, 19). However, MAP3K7 can serve as a tumor promoter or suppressor in liver cells, which is still controversial (30). Thus, we selected MAP3K7 for further validation of its effects on colony formation using transient transfection with siRNA (Figure 1D). Transient gene silencing of MAP3K7 significantly suppressed colony formation in SK-HEP-1 and Huh7 cells. Furthermore, knockdown of MAP3K7 had little effect on G_1_ phase arrest in cell cycle of SK-HEP-1 and Huh7 cells (Figure S1).

### MAP3K7 Kinase Is Involved in HCC Cell Migration and Invasion

**Figure 2:**
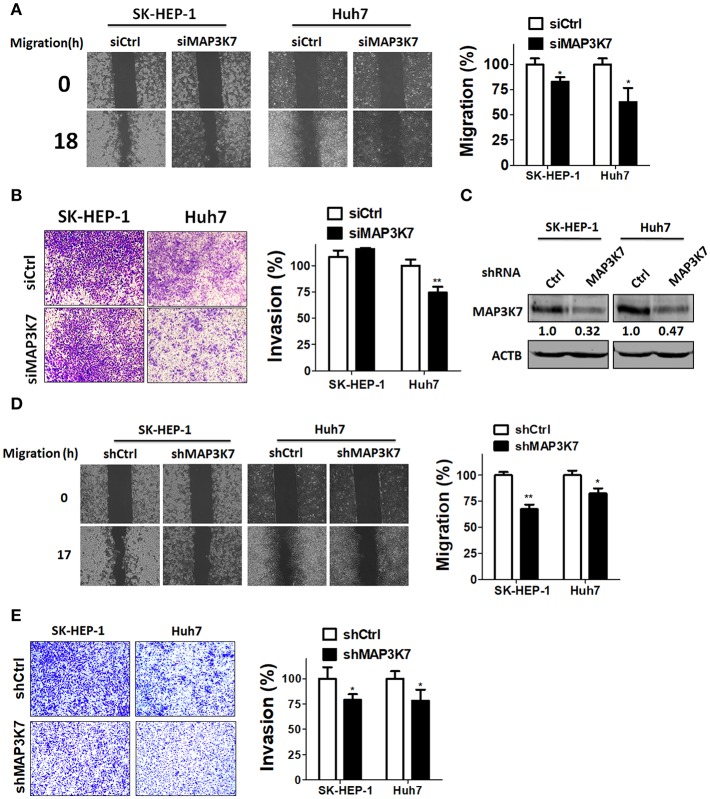
Effects of MAP3K7 on HCC cell migration and invasion. **(A)** Scramble siRNA (5 nM) or siRNA against MAP3K7 was transfected into SK-HEP-1 and Huh7 cells for 48 h. A cell migration assay was performed using a culture insert for control and MAP3K7-silenced HCC cells (left panel). The migratory distance of HCC cells was quantified with ImageJ software in the right panel. **(B)** The invasion of HCC cells transfected with scramble or siRNA against MAP3K7 was examined with Matrigel-coated Transwell filters. The cell invasion results were quantified with ImageJ software in the right panel. **(C)** Stable knockdown efficiency of MAP3K7 in SK-HEP-1 and Huh7 was confirmed by immunoblotting. **(D)** A cell migration assay and **(E)** invasion assay for stably knocked down HCC cells were performed. The quantified results for migration **(D)** and invasion **(E)** are showed in the right panel as the mean ± SEM from three independent experiments. ^*^p < 0.05; ^**^p < 0.01.

**Figure 3:**
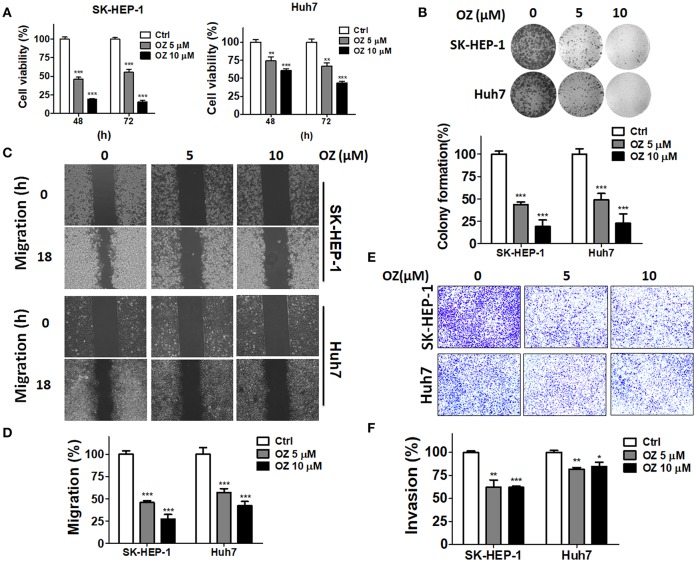
Effects of a MAP3K7 inhibitor on the cytotoxicity, migration, and invasion of HCC cells. **(A)** SK-HEP-1 and Huh7 cells were treated with the MAP3K7 inhibitor (5Z)-7-oxozeaenol for 48 or 72 h to measure cell proliferation with CellTiter Glo and **(B)** colony formation. **(C)** The cells were incubated overnight in a culture insert, and the insert was removed to assess cell migration in the presence or absence of (5Z)-7-oxozeaenol. **(E)** The effects of (5Z)-7-oxozeaenol on the invasion of HCC cells were determined by Matrigel-coated Transwell filters. The quantified results for migration **(D)** and invasion **(F)** are expressed as the mean ± SEM from three independent experiments. ^*^p < 0.05; ^**^p < 0.01; ^***^p < 0.001.

To examine if MAP3K7 is involved in the metastatic characteristics of SK-HEP-1 and Huh7 cells, these HCC cell lines were transfected with siRNA against MAP3K7 for migration and invasion assays. Migration was significantly attenuated in MAP3K7-silenced HCC cells compared to cells transfected with scramble siRNA (Figure 2A). Transient knockdown of MAP3K7 inhibited the invasion ability of Huh7 cells (Figure 2B). Similar to the results of transient knockdown, stable knockdown of MAP3K7 significantly inhibited the migration and invasion of HCC cells (Figures 2D,E). To evaluate further whether kinase activity is involved in cancer malignancy, HCC cells were treated with the MAP3K7 inhibitor (5Z)-7-oxozeaenol to determine its effects on cell proliferation, colony formation, migration, and invasion (Figure 3). (5Z)-7-oxozeaenol significantly reduced the growth and mobility of HCC cells. We further inspected if MAP3K7 is required for migration and invasion in HBV-positive HCC cells as well. Interestingly, silencing MAP3K7 had no effects on cell invasion in HBV-positive HA22T and Hep3B cell lines, while it promoted cell migration in Hep3B cells (Figure S2). In addition, treatment of (5Z)-7-oxozeaenol reduced migration and invasion in HA22T, whereas it increased cell invasion in Hep3B cells (Figure S3). These results suggested that MAP3K7 might be important for metastatic characteristics in certain types of HCC cells.

### Ablation of MAP3K7 Reduces Tumor Formation in Spheroid Cell Culture and xenografted Tumors

**Figure 4:**
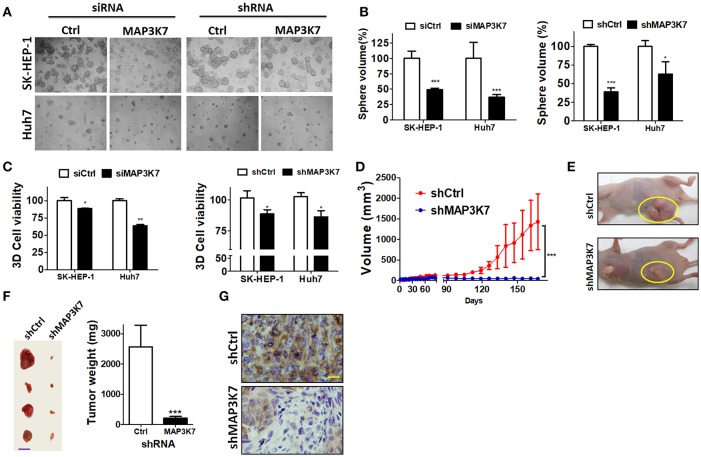
Effects of silencing MAP3K7 on tumor formation of HCC cells in spheroid culture and xenograft mouse models. **(A)** SK-HEP-1 and Huh7 cells harboring siRNA or shRNA against MAP3K7 were grown on NanoCulture plates for 7 days to observe spheroid formation under a microscope. **(B)** Sphere volume was calculated from at least 100 spheres in each condition and normalized by cells with scramble shRNA. **(C)** Cell proliferation in the spheres was measured by a 3D CellTiter Glo assay and is expressed as a percentage compared with control cells. **(D)** Stably knocked down SK-HEP-1 cells were xenografted into nude mice, and tumor size was monitored every 3–4 days (*n* = 5). **(E)** Representative images of xenograft tumors are shown. **(F)** The tumors were dissected from sacrificed mice and weighed. Representative tumors and quantitative results are shown. Scale bar: 20 mm. **(G)** MAP3K7 expression in xenograft tumors was confirmed by immunohistochemistry using an anti-MAP3K7 antibody. Scale bar: 50 μm. ^*^p < 0.05; ^**^p < 0.01; ^***^p < 0.001.

To assess precisely the effects of MAP3K7 in tumors, MAP3K7 was transiently or stably knocked down with siRNA and shRNA in HCC cells, respectively. The cells were seeded in NanoCulture plates to grow as spheroids to mimic tumor formation *in vivo* (Figure 4A). In contrast to cells transfected with scramble control, sphere volume and cell proliferation were significantly decreased in MAP3K7-silenced HCC cells (Figures 4B,C). To inspect further the effects of MAP3K7 in tumor formation *in vivo*, SK-HEP-1 cells stably harboring scramble shRNA or shRNA against MAP3K7 were injected subcutaneously into nude mice (Figure 4D). The volume of the xenografted tumors was measured and clearly showed that the growth of MAP3K7-knockdown HCC cells was much slower than that of control cells (Figures 4D,E). Likewise, tumor weight was significantly lower in MAP3K7-silenced HCC tumors (Figure 4F). The knockdown efficiency of MAP3K7 in xenografted tumors was confirmed by immunohistochemistry. The level of MAP3K7 protein was reduced in MAP3K7-silenced tumors (Figure 4G), confirming that HCC cells lacking MAP3K7 exhibited arrested tumor growth *in vivo*.

### MAP3K7 Is Associated With Tumorigenesis and Poor Prognosis in Patients With HCC

**Figure 5:**
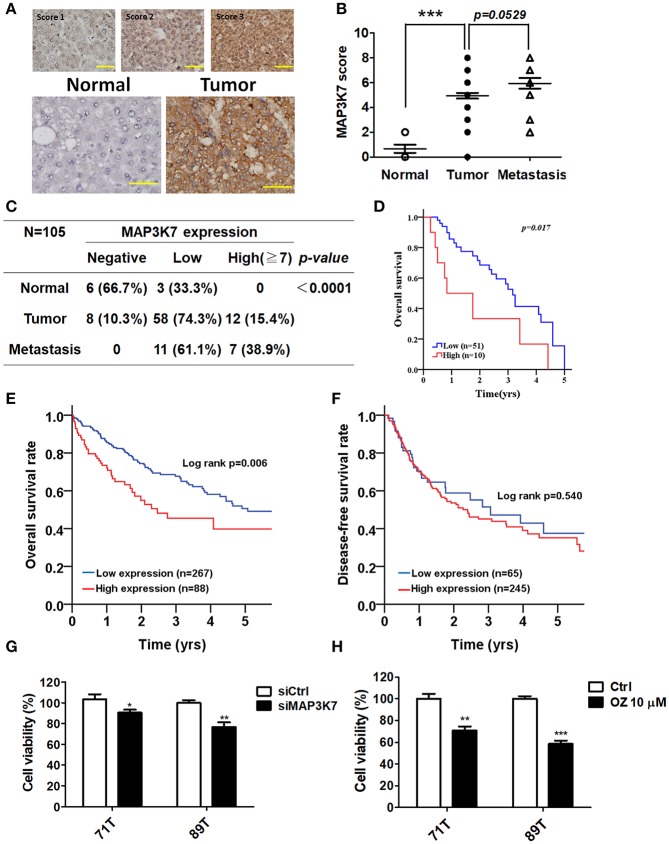
Clinical association of MAP3K7 expression with tumorigenesis and malignancy in patients with HCC. **(A)** MAP3K7 protein level in HCC tumor tissues was determined by immunohistochemistry, and the intensity score was categorized into three grades according to MAP3K7 expression (upper panel). Representative staining for MAP3K7 in adjacent normal tissues and tumor tissues is shown (upper panel). Scale bar: 100 μm. **(B)** The Allred score system was used to evaluate MAP3K7 protein levels in each tissue based on the sum of an intensity score and a proportion score ranging from 2 to 8. The quantitative results for MAP3K7 protein level in adjacent normal, tumor, and metastatic tumor tissues are shown. **(C)** MAP3K7 protein levels <5 and ≥5 were considered low and high expression, respectively. A protein level of 0 was defined as negative expression. *P*-values were estimated by the Wilcoxon-signed rank test. **(D)** Kaplan-Meier plots were used to analyze the association of MAP3K7 expression with overall survival. **(E)** The correlation of MAP3K7 gene expression with overall survival or **(F)** disease-free survival was analyzed according to an HCC dataset from TCGA. **(G)** The MAP3K7 gene was silenced in primary HCC cells by siRNA for 72 h, and the effects of silencing on cytotoxicity were determined with Cell-Titer Glo. **(H)** Primary HCC cells were treated with the MAP3K7 kinase inhibitor (5Z)-7-oxozeaenol for 24 h, and its cytotoxic effects were examined. ^*^p < 0.05; ^**^p < 0.01; ^***^p < 0.001.

MAP3K7 was found to promote cell growth and metastatic characteristics in cell culture models. To determine whether MAP3K7 expression is correlated with tumor development in patients with HCC, a tissue microarray containing tumor adjacent normal, tumor, and metastatic tissues was used to examine MAP3K7 expression with immunohistochemistry (Figure 5A). The level of MAP3K7 protein was higher in tumor tissues than in tumor adjacent normal tissues (*p* < 0.0001), while the level of MAP3K7 protein was slightly higher in metastatic tumor tissues than in primary tumor tissues (Figures 5B,C). High MAP3K7 protein level in tumor tissues had poor overall survival (Figure 5D). We further analyzed MAP3K7 expression levels in the HCC dataset obtained from TCGA. High MAP3K7 expression was associated with shorter overall survival, whereas it had no effect on disease-free survival (Figures 5E,F). To inspect the role of MAP3K7 on cancer cell survival, primary HCC cells were transfected with scramble siRNA or siRNA against MAP3K7. Silencing of MAP3K7 and the MAP3K7 inhibitor reduced the proliferation of primary HCC cells, including 71T (HBV-negative, HCV-positive) and 89T (HBV-positive, HCV negative) cells (Figures 5G,H).

### MAP3K7 Is Correlated With mTOR Expression and Associated With Poor Prognosis

**Figure 6:**
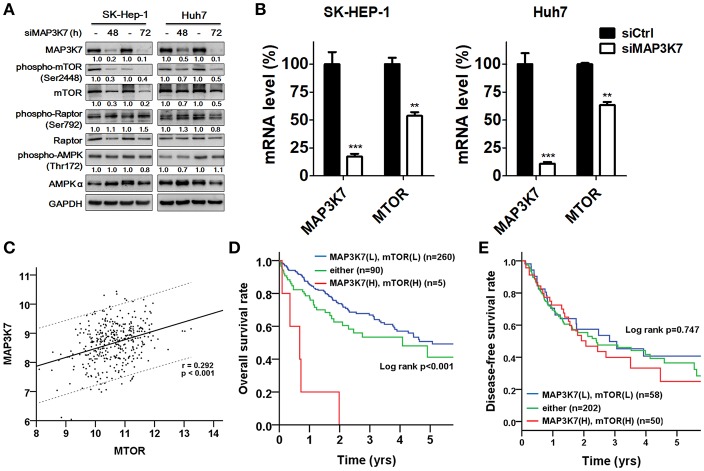
Correlation of MAP3K7 and mTOR in HCC. **(A)** SK-HEP-1 and Huh7 cells were transfected with scramble siRNA or siRNA against MAP3K7 for 48 or 72 h. The effects of silencing MAP3K7 on mTOR and p-(S2448)-mTOR were examined by immunoblotting using GAPDH as a control. **(B)** The gene expression of mTOR in MAP3K7 knockdown cells was determined by real-time PCR. **(C)** TCGA HCC dataset (*n* = 355) was used to analyze the correlation between MAP3K7 and mTOR expression in tumor tissues through the Pearson χ^2^ test. **(D)** Kaplan-Meier analysis was used to evaluate the effect of the co-expression of MAP3K7 and mTOR on overall survival and **(E)** disease-free survival. ^**^p < 0.01; ^***^p < 0.001.

MAP3K7 requires TAK1-binding protein 1 (TAB1), TAB2, and TAB3 to trigger NF-κB activation, which is a key transcription factor for tumor initiation and malignancy (31). Indeed, MAP3K7 knockdown attenuated the effects of tumor necrosis factor (TNF)-α stimulation on the transcriptional activity of NF-κB in SK-HEP-1 and Huh7 cells (data not shown). The correlation between MAP3K7 and TABs expression with the overall survival of patients with HCC was also inspected (Table S1). High co-expression of MAP3K7 and TAB1 was associated with shorter survival (Table S1, adjusted hazard ratio [AHR] = 2.23, *p* = 0.008). Similar results were observed in patients with high co-expression levels of MAP3K7 and TAB2 or TAB3 (TAB2: AHR = 1.79, *p* = 0.019; TAB3: AHR = 2.15, *p* = 0.002). Nevertheless, since these results have been reported previously in cancer cells (31) and MAP3K7 was selected from our screen in regular medium without TNF-α stimulation, we set them aside. Moreover, MAP3K7 inactivation induces AMPK activity and diminishes phosphorylation of MTOR in skeletal muscle cells (32). Thus, we inspected the involvement of the potential downstream effector mTOR in MAP3K7-modulated tumor malignancy in HCC cells. Interestingly, knockdown of MAP3K7 decreased mTOR phosphorylation and protein level without affecting AMPK phosphorylation in HCC cells (Figure 6A). The expression of mTOR mRNA was also decreased in MAP3K7-silenced HCC cells (Figure 6B). TCGA data analysis showed that MAP3K7 expression was positively correlated with mTOR levels (Figure 6C). The association of survival with MAP3K7 expression alone or combined with mTOR expression was evaluated by univariate and multivariate Cox proportional hazards models (Table 1). High MAP3K7 expression was associated with poor overall survival in patients with HCC (AHR = 1.78, *p* = 0.006). Co-expression with either MAP3K7(High)/mTOR(Low) or MAP3K7(Low)/mTOR(High) was associated with shorter overall survival (AHR = 1.52, *p* = 0.036), while co-expression of MAP3K7(High)/mTOR(High) had much worse overall survival (AHR = 8.26, *p* < 0.001). However, neither MAP3K7 expression alone nor in combination with mTOR expression correlated with disease-free survival. Similarly, Kaplan-Meier plots showed that high co-expression of MAP3K7 and mTOR was associated with significantly shorter overall survival (Figure 6D), whereas it had no correlation with disease-free survival (Figure 6E), suggesting that the MAP3K7/mTOR axis is involved in tumor malignancy, but not recurrence, in patients with HCC.

**Table 1:** Co-expression of MAP3K7 and MTOR in overall survival and disease-free survival of HCC patients.

**Variable**		**No. (%)**	**CHR (95% CI)**	***p*-value**	**AHR (95% CI)**	***p*-value**
**OVERALL SURVIVAL**						
MAP3K7	Low	267 (75.2)	1.00		1.00	
	High	88 (24.8)	1.81 (1.24–2.64)	0.002^*^	1.78 (1.18–2.69)	**0.006**^‡^
MTOR	Low	343 (96.6)	1.00		1.00	
	High	12 (3.4)	1.93 (0.90–4.13)	0.093^*^	1.84 (0.76–4.47)	0.177^‡^
MAP3K7 (L) MTOR (L)		260 (73.2)	1.00		1.00	
Either		90 (25.4)	1.44 (0.98–2.12)	0.065^*^	1.52 (1.03–2.24)	**0.036**^**†**^
MAP3K7 (H) MTOR (H)		5 (1.4)	7.37 (2.97–18.27)	< 0.001^*^	8.26 (3.31–20.65)	**< 0.001**^**†**^
**DISEASE-FREE SURVIVAL**						
MAP3K7	Low	65 (21.0)	1.00		1.00	
	High	245 (79.0)	1.14 (0.75–1.74)	0.540^*^	1.20 (0.77–1.88)	0.428^‡^
MTOR	Low	253 (81.6)	1.00		1.00	
	High	57 (18.4)	1.12 (0.74–1.70)	0.600^*^	1.33 (0.86–2.05)	0.200^‡^
MAP3K7 (L) MTOR (L)		58 (18.7)	1.00		1.00	
Either		202 (65.2)	1.03 (0.72–1.46)	0.891^*^	1.14 (0.72–1.79)	0.576^†^
MAP3K7 (H) MTOR (H)		50 (16.1)	1.12 (0.73–1.74)	0.599^*^	1.24 (0.71–2.18)	0.449^†^

*SCC, squamous cell carcinoma; CHR, crude hazard ratio; CI, confidence interval; AHR, adjusted hazard ratio*.

* *p-values were estimated by Cox's regression*.

† *p-values were estimated by multivariate Cox's regression*.

‡ *p values were adjusted for cell differentiation (moderate+poor vs. well) and AJCC pathological stage (stage III+IV vs. stage I+II) by multivariate Cox's regression. Significant p-value (< 0.05) was marked as bold*.

## Discussion

Targeted therapy is a type of treatment that blocks specific oncogenic molecules to diminish cancer growth and invasiveness, such as, trastuzumab (Herceptin) for human epidermal growth factor receptor 2 in breast cancer (33), imatinib (Gleevec) for BCR-Abl in chronic myelogenous leukemia (34), and bevacizumab (Avastin) for vascular endothelia growth factor (VEGF) in colorectal and lung cancer (35). These results indicate that many kinases are associated with tumor development and malignancy in various cancer types (36). Sorafenib inhibits multiple kinases, including Raf, VEGFR, and PDGFR, but it has limited benefit in a few populations of HCC patients (37). Moreover, no single kinase is reported to be an effective target for patients with HCC. Herein, we comprehensively screened two HCC cell lines with a kinome siRNA library for potential therapeutic targets for HCC. Our study reported the following findings. First, genetic and pharmacological ablation of MAP3K7 attenuated HCC cell growth in two-dimensional and spheroid cell culture, as well as in xenografted tumors. Second, deprivation of MAP3K7 inhibited the migration and invasion of HCC cells. Third, MAP3K7 expression was higher in tumor tissues, and high MAP3K7 expression, alone or in combination with its downstream regulator mTOR, was associated with poor survival in patients with HCC.

Through kinome-wide screening for potential targets of HCC with an siRNA library, knockdown of several of the identified kinases sufficiently blocked cell proliferation in various HCC cell lines, including HBV-positive and -negative HCC cell lines. Spheroid cell culture results also showed that silencing MAP3K7 inhibited sphere formation in HBV-negative (SK-HEP1, Huh7, and 71T) and HBV-positive (89T) cells. Moreover, SK-HEP-1 cells have wild-type TP53, whereas Huh7 cells have mutant TP53 (38). The TP53 gene was deleted in the other two cell lines, Hep3B and HA22T. Silencing MAP3K7 diminished cell growth in HCC cell lines without or with wild-type or mutant TP53, implying that the effect of MAP3K7 on cell proliferation might be in an HBV- and TP53-independent manner. More interestingly, gene knockdown of some kinases showed stronger inhibitory effects in SK-HEP-1 cells than in TP53 mutant (Huh7) and deleted (Hep3B and HA22T) cells, such as YES, ABL1, and STK17B. The results suggested that TP53 inactivation might be involved in kinase-mediated tumorigenesis, which warrants further investigation. On the other hand, the opposite effects of MAP3K7 in migration and invasion were observed in Hep3B cells, but not HA22T cells. The results suggested that some other factors may interfere MAP3K7 signaling on cell migration and invasion, which require further study to elucidate.

In addition to MAP3K7, nuclear receptor binding protein 2 (NRBP2), PLK1, AURKA, and PIK3CA have been reported to be important in HCC. AURKA and PLK1 expression levels are elevated in tumor tissues compared to adjacent normal tissues in patients with HCC (19, 39). Silencing PLK1 arrests the cell cycle at the G2/M phase and induces apoptosis (39), while knockdown of AURKA reduces the post-radiotherapy invasion of HCC (19). Moreover, PIK3CA is activated by a mutation in 28% of HCC patients (40). PI-103 (an inhibitor of PI3K and mTOR) inhibits cell proliferation and enhances the chemosensitivity of HCC cells to sorafenib (41). These results suggest that our screening platform could be used to find new therapeutic targets for HCC when screening other siRNA libraries. In contrast, NRBP2 expression is lower in tumor tissues, and high NRBP2 expression is associated with better prognosis and chemosensitivity to sorafenib (42), suggesting that it serves as a tumor suppressor. Nevertheless, our current data showed that both transient and stable knockdown of NRBP2 diminished the proliferation of HCC cells, which requires further study to elucidate its role in HCC.

MAP3K7 is a member of the MAP3KKK family and is also known as transforming growth factor (TGF)-β1-activated kinase (TAK1), which is modulated by TGF-β and several cytokines, such as TNF-α, interleukin-1 (IL-1), Toll-like receptors, CD40, and B cell receptors (31). These cytokines induce the formation of the activated MAP3K7 complex via TNF receptor association factor 2 (TRAF2) and TRAF6 (30). TAB1 and TAB2/3 associate with the N-terminus and C-terminus, respectively, of MAP3K7 for its full activation (43, 44). MAP3K7 interacts with IκB kinase (45) and MAPK to enhance the transcriptional activity of NF-κB and AP-1, which are associated with inflammation and tumorigenesis. Gene silencing of MAP3K7 enhances the sensitivity of cancer cells, but not normal epithelial cells, to chemotherapeutic drugs (46). The MAP3K7 inhibitor LYTAK1 blocks NF-κB activity to increase chemotherapeutic efficacy in pancreatic cancer cells (47). Conversely, MAP3K7 conditional knockout in liver parenchymal cells of mice induces hepatocyte dysplasia and early-onset hepatocarcinogenesis (48). In our present study, MAP3K7 expression was much higher in tumor tissues than in adjacent normal tissues and was associated with poor survival of HCC patients. Gene silencing of MAP3K7 blocked HCC cell growth in cell culture and a tumor xenograft mouse model, suggesting that low levels of MAP3K7 may be essential for the normal physiological function of hepatocytes, but too much MAP3K7 may cause inflammation and promote HCC, particularly in cytokine-elevated conditions.

In addition to cytokines, MAP3K7 can be activated by various viruses (49, 50), which are also known as high-risk factors for hepatocarcinogenesis in HCC, including HBV and hepatitis C virus (HCV). The X protein of HBV can activate IKK to activate mTOR and the downstream effector S6K1 (51). HCV infection increases IKK-α expression for lipogenesis (52), and its core protein triggers the activation of NF-κB for the gene expression of the inflammatory cytokine IL-1β through TRAF2/6 (53), which are crucial factors for activated MAP3K7 complex formation. However, the missing links between HBV/HCV and MAP3K7 activation for HCC development need further study to be elucidated. Moreover, MAP3K7 reportedly phosphorylates mTOR to regulate mitochondrial function (32). mTOR is overexpressed in approximately 50% of HCC patients and is associated with poor differentiation and prognosis (20). mTOR inhibitors are currently being tested in clinical trials for patients with HCC (NCT10687673 and NCT01177397). Our present data showed that silencing MAP3K7 not only reduced the phosphorylation of mTOR but also diminished its protein and mRNA levels. High co-expression levels of MAP3K7 and mTOR were significantly associated with poor overall and disease-free survivals in patients with HCC compared to those with low expression levels. Nevertheless, the patients' number (*n* = 5) with high co-expression levels of MAP3K7 and MTOR in overall survival is not large enough as shown in Table 1. Our study may need a larger cohort of HCC to verify the association of these genes in overall survival.

In addition, MAP3K7 decreases AMPK activity, whereas it increases phosphorylation of both p38 and MTOR (32). AMPK can phosphorylate regulatory-associated protein of mTOR (raptor) at Ser792 to inactivate MTOR. Our current results showed that silencing MAP3K7 reduced MTOR phosphorylation without changing phosphorylation of AMPK and raptor. P38 is involved in MTOR activation in cardiomyocyte (54). It raises a possibility that MAP3K7 may regulate MTOR through p38 activation. Although the molecular mechanisms by which MAP3K7 affects mTOR expression require further study, these results indicate that MAP3K7 might be a potential diagnostic marker or therapeutic target for future drug development in HCC.

## Ethics Statement

This study was carried out and approved by the Institutional Animal Care and Use Committee at Kaohsiung Veterans General Hospital.

## Author Contributions

J-SC, W-LT, P-FL, and C-WL performed most of the experiments, with the exception of the experiments performed by H-HT (Figures 2B–D) and Y-GG (Figures 2E,3E–F). Tumor xenograft mouse experiments were performed by C-HL. C-WS prepared the figures, performed the statistical analyses, interpreted the data, and wrote the manuscript.

### Conflict of Interest Statement

The authors declare that the research was conducted in the absence of any commercial or financial relationships that could be construed as a potential conflict of interest.

## Abbreviations

MAP3K7: mitogen-activated protein kinase kinase kinase 7
HCC: hepatocellular carcinoma
mTOR: mammalian target of rapamycin
TGF-β1: transforming growth factor beta 1.
