# Supplementary material for: The MAP3K7-mTOR Axis Promotes the Proliferation and Malignancy of Hepatocellular Carcinoma Cells
Source: Front Oncol. 2019 Jun 4;9:474. doi: 10.3389/fonc.2019.00474 (PMC6558008; doi:10.3389/fonc.2019.00474)
Supplement: Supplementary file 1 [file Data_Sheet_1.PDF]

Table S1. The co-expression levels of MAP3K7 and TABs in overall survival of HCC patients

Variable (Cheng et al.)	No. (%)	CHR (95% CI)	*p*-value	AHR (95% CI)	*p*-value *
**MAP3K7XTAB1**					
MAP3K7(L), TAB1(L)	51 (14.4)	1		1	
either	234 (65.9)	0.79 (0.55-1.12)	0.186	1.26 (0.74-2.16)	0.400
MAP3K7(H), TAB1(H)	70 (19.7)	1.85 (1.25-2.74)	**0.002**	2.23 (1.23-4.06)	**0.008**
MAP3K7XTAB2					
MAP3K7(L), TAB2(L)	94 (26.5)	1		1	
either	181 (51.0)	0.77 (0.54-1.09)	0.135	1.01 (0.65-1.57)	0.959
MAP3K7(H), TAB2(H)	80 (22.5)	1.78 (1.21-2.62)	**0.004**	1.79 (1.10-2.92)	**0.019**
MAP3K7XTAB3					
MAP3K7(L), TAB3(L)	114 (32.1)	1		1	
either	172 (48.5)	0.95 (0.67-1.34)	0.749	1.29 (0.85-1.96)	0.240
MAP3K7(H), TAB3(H)	69 (19.4)	1.84 (1.24-2.73)	**0.002**	2.15 (1.33-3.45)	**0.002**

Abbreviations: SCC, squamous cell carcinoma; CHR, crude hazard ratio; CI, confidence interval; AHR, adjusted hazard ratio.
**p-value were adjusted by multiple Cox's regression.*
Bold values denote statistically significant

SUPPLEMENTARY FIGURE

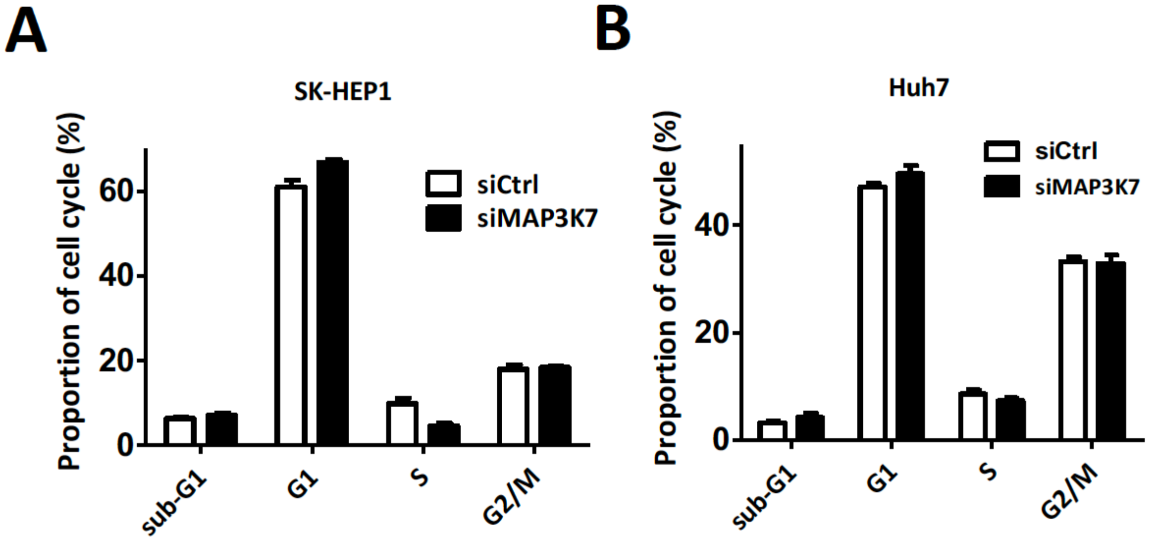

Figure S1. The effects of MAP3K7 on cell cycle proportion in HCC cells.

(A) SK-HEP-1 and (B) Huh7 cells were transfected with scramble siRNA or siRNA against MAP3K7 for 72 h. The cells were harvested and stained with propidium iodide for cell cycle analysis with flow cytometer. The effects of silencing MAP3K7 on cell cycle proportion were quantitated with FlowJo software.

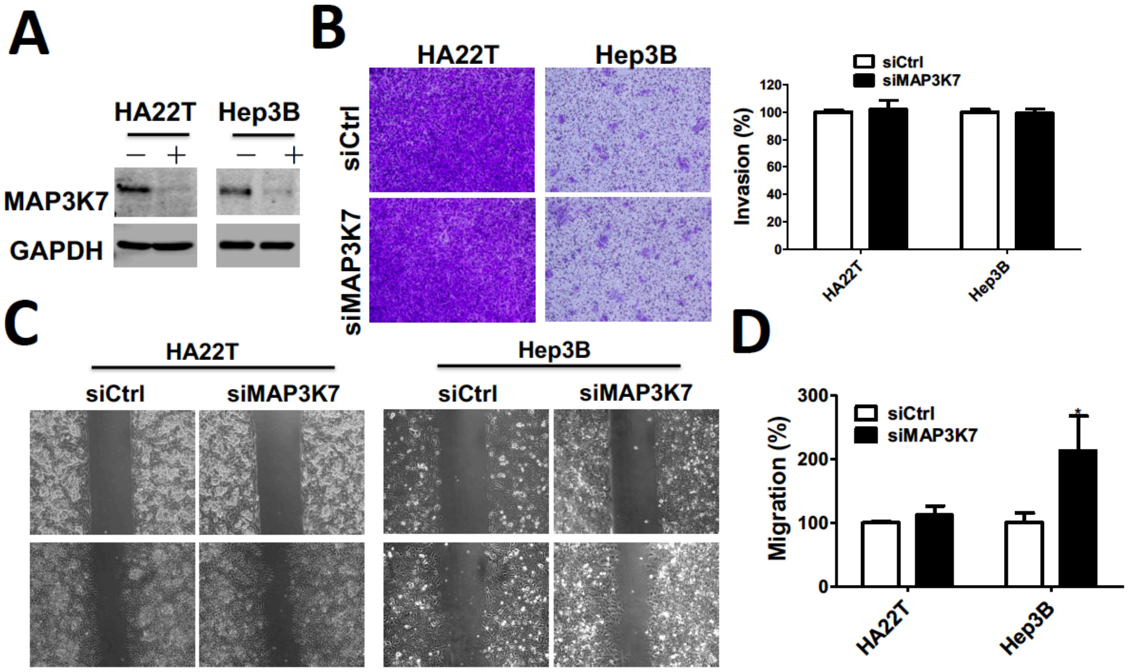

Figure S2. Effects of silencing MAP3K7 on migration and invasion in HBV-positive HCC cell lines

(A) Scramble siRNA (5 nM) or siRNA against MAP3K7 was transfected into HA22T and Hep3B cells for 72 h, and knockdown efficiency was confirmed by immunoblotting. (B) The invasion of HCC cells transfected with scramble or siRNA against MAP3K7 was examined with Matrigel-coated Transwell filters. The cell invasion results were quantified with ImageJ software in the right panel. (C) A cell migration assay was performed using a culture insert for control and MAP3K7-silenced HCC cells. (D) The migratory distance of HCC cells was quantified with ImageJ software. The quantified results for migration and invasion are expressed as the mean ± SEM from three independent experiments.

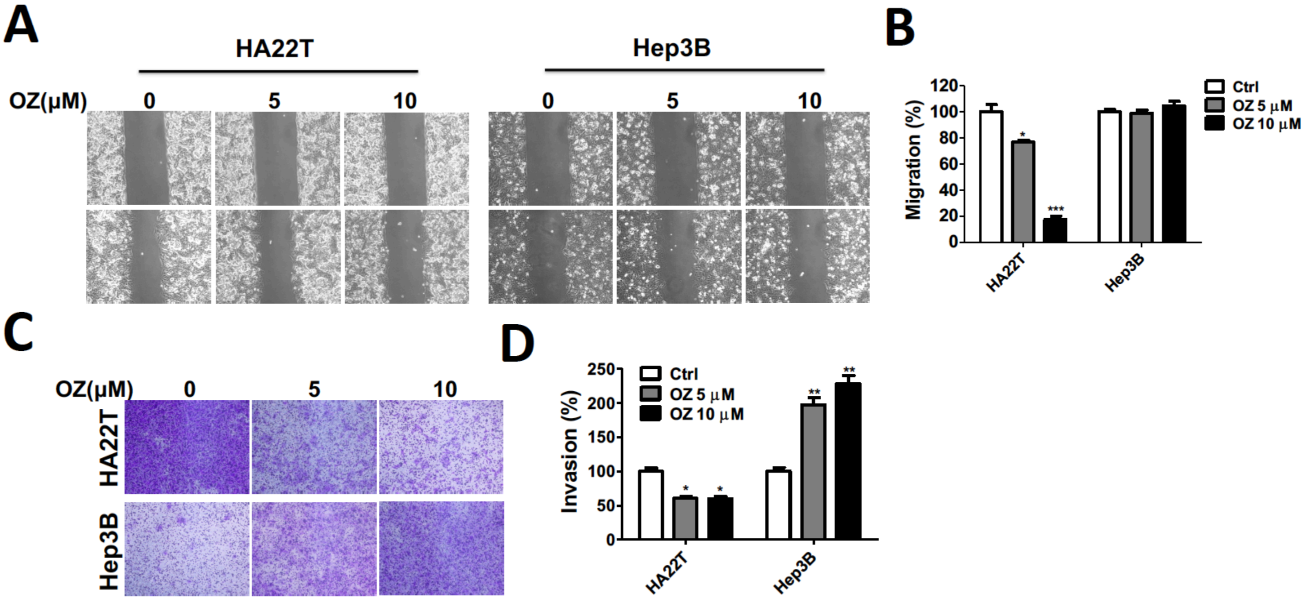

Figure S3. Effects of a MAP3K7 inhibitor on the migration and invasion in HBV-positive HCC cell lines

(A) HA22T and Hep3B cells were incubated overnight in a culture insert, and the

insert was removed to assess cell migration in the presence or absence of (5Z)-7-oxozeaenol (OZ). (C) The effects of (5Z)-7-oxozeaenol on the invasion of HCC cells were determined by Matrigel-coated Transwell filters. The quantified results for migration (B) and invasion (D) are expressed as the mean ± SEM from three independent experiments.
